# Short implants in the posterior maxilla to avoid sinus augmentation procedure: 5-year results from a retrospective cohort study

**DOI:** 10.1186/s40729-018-0155-1

**Published:** 2019-01-22

**Authors:** Jonas Lorenz, Maximilian Blume, Tadas Korzinskas, Shahram Ghanaati, Robert A. Sader

**Affiliations:** 1 FORM-Lab, Department for Oral, Cranio-Maxillofacial, and Facial Plastic Surgery, Medical Center of the Goethe University Frankfurt, Frankfurt am Main, Germany; 2 Private Dental Practice, Mainz, Germany; 3 Private Practice, Bokštų 9, LT-92125 Klaipeda, Lithuania

**Keywords:** Dental implants, Short implants, Camlog, Clinical study, Marginal bone loss

## Abstract

**Background:**

Short implants present a promising approach for patients with advanced atrophy to avoid augmentative procedures. However, concerns about increased biological and technical complications due to an unfavorable implant-crown ratio are still present.

**Purpose:**

The aim of the present retrospective study was to evaluate whether a reduced implant length has any impact on implant success and peri-implant hard and soft tissue health in implants placed in the posterior maxilla to avoid sinus augmentation procedures.

**Materials and methods:**

Fourteen patients received a total of 30 implants of 7-mm length in the posterior maxilla. Implants with a mean loading period of 5 years (range 2–7 years) were followed up clinically and radiologically, with a focus on the peri-implant soft tissue parameters probing pocket depth (PPD), bleeding on probing (BoP), and the stability of the marginal peri-implant bone level.

**Results:**

None of the implants were lost, and no technical failures occurred. A mean PPD of 2.5 mm, a mean BoP of 13.3%, and a mean marginal bone loss (MBL) of 0.5 mm indicate healthy peri-implant hard and soft tissue conditions without signs of peri-implantitis.

**Discussion:**

The present results indicate the suitability of implants of 7-mm length to replace missing teeth in the posterior maxilla. An unfavorable implant-crown ratio or reduced bone-implant contact length seems to have no negative influence on midterm implant success or on peri-implant hard and soft tissue health.

## Introduction

In the past few decades, technical developments of dental implants in combination with continuous development of surgical techniques and biomaterials have led to an expansion of the indications for implant-retained prosthetics. Prevention of atrophy after tooth extraction by socket or ridge preservation or reconstruction of the alveolar crest in cases of atrophy by augmentation with autologous bone or bone substitute materials of different origins have become reliable treatment options to establish a sufficient implantation bed [1–4].

However, extensive augmentation procedures as therapy of choice for all patients should be viewed critically. Due to compromised general health, anamnestic data, or individual demands of the patient, minimally invasive methods to restore oral function should be considered. In this context, the development and scientific investigation of so-called short implants, which are implants with reduced length, play an important role, as they seem to allow placement of dental implants in the molar region of the atrophic maxilla and, at the same time, avoid the need for sinus augmentation procedures. A further indication of short implants is the molar region of the lower jaw, in which the possibility of implant placement can be restricted due to the anatomical position of the nervus alveolaris inferioris.

Although short implants have been reported in the literature for several years, the term “short implants” is used quite heterogeneously to indicate implant lengths. While in the present study short implants with a length of 7 mm are investigated, Mangano et al. considered short implants to have a length of 8 mm [5]. This guiding value has also been reported as 8.5 mm, and even 10 mm, which indicates scientific disagreement on this topic [6, 7].

Major concerns regarding technical and biological complications due to the increased crown-implant ratio in short-length implants have been expressed. In a systematic review, Blanes et al. excluded correlation between the occurrence of biological and technical complications and the crown-implant ratio of implant-supported reconstructions [8]. Especially regarding bone loss, the literature review showed that the crown-implant ratio does not influence peri-implant crestal bone loss. Similar findings are reported by Nunes et al., who reported that dental implants of 4-mm width and 7-mm length and implant-supported fixed prostheses with a crown-implant ratio larger than 2 had no positive correlation to marginal bone loss [9].

In the present retrospective study, implants of 7-mm length and a specific implant design, including a conical implant-abutment connection and platform switching, placed in the posterior maxilla were investigated by means of a clinical and radiological analysis after a mean loading period of 5 years. The aim of this study was to analyze whether a reduced implant length has any impact on implant success and peri-implant hard and soft tissue health.

## Materials and methods

### Study design and patient population

In the present retrospective study, 14 patients (5 females and 9 males) with a mean age of 63 years (34–80 years) received Conelog® Screw-line implants (Camlog Biotechnologies, Basle, Suisse) with a length of 7 mm. In total, 30 implants were clinically and radiologically investigated after a mean loading period of 5 years (range 2–7 years).

All patients from the Department for Oral, Cranio-Maxillofacial and Facial Plastic Surgery, Medical Center of Goethe University Frankfurt who received implants of 7-mm length in the posterior maxilla to avoid a sinus augmentation procedure within the past 7 years were screened. Furthermore, the implants had to be loaded for at least 2 years. The initial residual bone height varied between 6 and 8 mm, leading to a bicortical fixation of the implants. The kind of prosthetic restoration (removable/fixed, splinted/un-splinted) was not defined as an inclusion/exclusion criteria. Patients with incomplete data collection and those who refused to participate in the study were excluded. A total of 17 patients met the inclusion criteria, 14 of whom were available for follow-up investigation and were included in the present study.

The study was approved by the ethics commission of the medical department of Goethe University in Frankfurt am Main, Germany (79/18), and it was conducted according to the fifth revision of the World Medical Association Declaration of 2000 (version, 2008). All participating patients gave informed written consent to participate in the retrospective study and for publication of the obtained data.

**Fig. 1:**
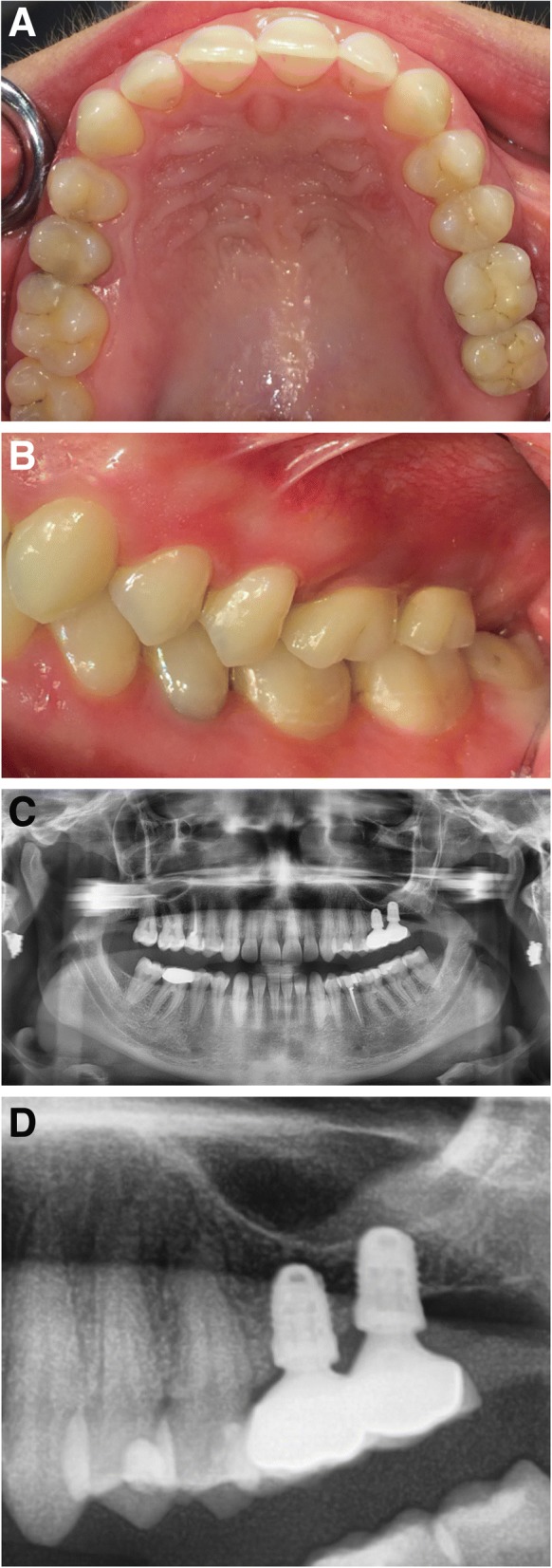
The clinical images of patient 4, with implant-supported single crowns in regions 26 and 27. No signs of a peri-implant infection, mucositis, peri-implantitis, or marginal bone loss were detected. **a** Occlusal view. **b** Left-side view. **c** Orthopantogram. **d** Close-up radiographic view

All implants were placed at least 3 months after the extraction of teeth in the posterior maxilla that were not able be preserved. If necessary, minor guided bone regeneration (GBR) procedures were performed simultaneously with the implant placement using a xenogenous bone substitute material (Bio-Oss®, Geistlich Biomaterials, Wolhusen, Suisse). After a mean healing period of 4 months (range 4–5 months), prosthetic rehabilitation was performed, which included fixed single-crown prosthetics in 21 implants and removable implant-retained dentures in 9 implants. Out of the 21 implants restored with single-crown prosthetics, only 4 crowns in 2 patients were splinted (patient no. 1 and patient no. 4, Fig. 1). All 9 implants retaining removable prosthetics have been restored with electroplated telescopic crowns and are therefore also not primary splinted.

After a mean loading period of 5 years (range 2–7 years), the implants were clinically and radiologically analyzed to determine the overall implant success, mean survival and suitability for prosthetic rehabilitation, peri-implant hard and soft tissue health, and patient acceptance. Furthermore, peri-implant hard and soft tissue indices, such as bleeding on probing (BoP), probing pocket depth (PPD), marginal bone loss (MBL), and presence of peri-implant osteolysis, were analyzed.

Table 1 gives an overview of the patient information, implant localization, and implant data for the retrospectively investigated implants.

**Table 1:** An overview of the patient information, implant localization, and implant data from the retrospectively investigated implants

Patient	Gender (m/f)	Age (years)	Implant-localization (region)	Implant-diameter (mm)	Implant-length (mm)	Prosthetic rehabilitation
1	m	52	26	4.3	7	f.p.
			27	4.3	7	f.p.
			16	4.3	7	f.p.
2	m	72	15	4.3	7	r.p.
3	f	73	15	4.3	7	r.p.
			25	4.3	7	r.p.
4	f	34	26	3.8	7	f.p.
			27	3.8	7	f.p.
5	m	73	14	3.8	7	f.p.
			15	3.8	7	f.p.
			25	3.8	7	f.p.
6	m	55	15	4.3	7	f.p.
			24	3.8	7	f.p.
7	f	83	24	3.8	7	r.p.
			26	4.3	7	r.p.
8	m	62	16	5	7	f.p.
			26	5	7	f.p.
9	m	67	16	5	7	r.p.
			26	5	7	r.p.
10	f	56	16	3.8	7	f.p.
			17	4.3	7	f.p.
11	m	74	23	3.8	7	r.p.
			24	4.3	7	r.p.
12	m	54	16	4.3	7	f.p.
			17	4.3	7	f.p.
			26	3.8	7	f.p.
13	f	56	16	4.3	7	f.p.
			17	5.0	7	f.p.
14	m	80	25	4.3	7	f.p.
Total/mean:	5*f; 9*m	63	30	14*4.3; 6*5.0; 10*3.8	30*7 mm	21*f.p., 9*r.p

*f* female, *m* male, *f.p.* fixed prosthetics, *r.p.* removable prosthetics, * e.g. 5 female and 9 male patients

### Analyzed implant system

In the present retrospective study, Conelog® Screw-line implants (Camlog Biotechnologies, Basle, Suisse) of 7-mm length and of diameters of 3.8 mm, 4.3 mm, and 5.0 mm were used to replace missing teeth in the molar region of the maxilla. The implant system has a Morse-locking conical implant-abutment connection with platform switching and 3-point indexing. The Promote® surface of the implant system is manufactured by grit blasting and acid etching that support osseointegration.

### Clinical and radiological follow-up investigation

After a mean period of 5 years (range 2–7 years), the patients were clinically and radiologically investigated at the Department for Oral, Cranio-Maxillofacial, and Facial Plastic Surgery of the Medical Center of Goethe University Frankfurt according to previously published methods [1, 3, 10].

The following parameters were investigated to determine the clinical suitability of the short-length implants and the peri-implant hard and soft tissue stability: implant survival, i.e., implants being in situ and suitable for prosthetic restoration; the width and thickness of the peri-implant keratinized gingiva (in mm); PPD (in mm); BoP; radiologically calculated MBL; and presence of peri-implant osteolysis. The PPD was measured at 4 sites (mesio-buccal, distal-buccal, mesio-oral, and disto-oral) with a blunt periodontal probe. Simultaneously, to the measurement of the probing pocket depths, the peri-implant soft tissue was checked to see if the probing provoked bleeding (BoP). The peri-implant MBL was calculated at digitally recorded perpendicular single-tooth images taken routinely after implant insertion and for the regular follow-up checks. The distance between the peri-implant marginal bone level and the implant shoulder serving as reference point was measured. Bone loss was measured mesially and distally, and a mean bone loss value from these measurements was calculated.

#### Investigation parameters

- Implant being in situ and suitable for prosthetic rehabilitation
- Buccal width and thickness of peri-implant keratinized gingiva
- Probing depth (at 4 sites per implant)
- BoP (per implant)
- Peri-implant bone loss
- Presence of peri-implant osteolysis

## Results

### Clinical results

After patient screening was performed, 30 implants in the premolar and molar regions of the upper jaw in 14 patients met the inclusion criteria and were clinically and radiologically followed up according to the study protocol. The aim of the follow-up investigation was to analyze whether implants of 7-mm length are suitable for prosthetic rehabilitation in the atrophic maxilla to avoid a sinus augmentation procedure.

After a mean loading period of 5-years, all 30 implants were in situ and suitable for prosthetic rehabilitation (survival rate of 100%). All implants were stable without signs of mobility. Twenty-one implants were restored with cement-retained fixed crowns, while 9 implants were restored with removable superstructures. Removable superstructures have been restored with electroplated telescopic crowns and have therefore not been primary splinted. Also, the majority of fixed superstructures (17) have been un-splinted single crowns, while only 4 implants in 2 patients have been restored with splinted single crowns. No major complications during healing and loading were recorded.

Analysis of the width and thickness of peri-implant keratinized gingiva indicated a mean peri-implant keratinized gingiva thickness of 1.8 mm (1–3 mm) and peri-implant keratinized gingiva width of 2.0 mm (1–3 mm). Gingival recessions of 1 mm at the facial contour were detected in 4 implants, which led to exposition of the implant shoulder.

Measurements of the PPD were recorded at four sites per implant (mesio-buccal, disto-buccal, mesio-oral, and disto-oral) and indicated a mean PPD of 2.5 mm (1–5 mm). The inflammatory conditions of the peri-implant tissue, which were analyzed by recording bleeding after measuring the PPD, indicated BoP in 4 of the 30 implants, so the BoP ratio was 13.3%. A distinct correlation between an increased PPD and increased BoP was shown, as the BoP was recorded at most implants presenting with PPDs of more than 4 mm. The entire clinical follow-up investigated revealed no impact of prosthetic restorations (fixed or removable, splinted or un-splinted prosthetics) on per-implant soft-tissue health.

### Radiological results

To analyze peri-implant bone loss over the study period of 5 years, digitally recorded perpendicular single-tooth images recorded immediately after implant placement and at the follow-up investigation were compared.

A mean total peri-implant marginal bone loss of 0.5 mm, ranging from 0 to 1.5 mm, was shown. Sub-analysis indicated mesial peri-implant bone loss of 0.4 mm and distal peri-implant bone loss of 0.6 mm. Furthermore, no signs of acute peri-implant infection or peri-implant osteolysis were presented, and no difference regarding prosthetic restoration could be observed (Table 2).

**Table 2:** An overview of the results of the clinical and radiological follow-up investigation

Patient	Implant-localization (region)	Implant-loss (+/−)	Buccal width of keratinized peri-implant gingiva (mm)	Buccal thickness of keratinized peri-implant gingiva (mm)	Probing depth (mm) at four sites (mb, db, mo, do)	Bleeding on probing (+/−) (per implant)	Marginal bone loss (mm) (mesially and distally)	Recession (mm)	Presence of peri-implant osteolysis (+/−)
1	26	−	2	2	3, 2, 2, 2	−	0, 0	–	−
	27	−	2	2	2, 3, 2, 3	−	0, 0	–	−
	16	−	3	3	2, 2, 2, 2	−	0, 0.5	–	−
2	15	−	1	1	3, 3, 2, 2	−	1, 1	–	−
3	15	−	2	3	2, 3, 2, 2	−	0, 0	–	−
	25	−	2	2	2, 2, 2, 2	−	0, 0	–	−
4	26	−	3	3	3, 2, 2, 2	−	0, 0.5	–	−
	27	−	2	2	3, 3, 2, 2	−	0.5, 0.5	–	−
5	14	−	3	2	2, 2, 3, 2	−	0, 0.5	–	−
	15	−	2	2	2, 3, 2, 2	−	0.5, 1	–	−
	25	−	2	2	2, 2, 1, 2	−	0, 0.5	–	−
6	15	−	3	2	1, 1, 2, 1	−	0, 0	–	−
	24	−	3	2	2, 2, 3, 3	−	0, 1	–	−
7	24	−	1	1	2, 3, 2, 2	−	0.5, 0.5	–	−
	26	−	1	1	3, 3, 3, 3	−	0.5, 0.5	–	−
8	16	−	2	2	3, 3, 4, 4	−	1, 1	–	−
	26	−	2	2	3, 4, 3, 4	−	1, 1	–	−
9	16	−	1	1	3, 4, 3, 3	−	0.5, 0.5	1	−
	26	−	1	1	3, 3, 3, 3	−	0.5, 0.5	1	−
10	16	−	2	2	3, 3, 4, 4	+	1, 1	–	−
	17	−	2	1	3, 3, 4, 3	+	1, 1.5	–	−
11	23	−	1	1	4, 3, 3, 5	+	1, 1	1	−
	24	−	1	1	5, 4, 2, 3	+	1, 1	1	−
12	16	−	2	2	2, 2, 2, 2	−	0, 0.5	–	−
	17	−	2	2	2, 3, 2, 2	−	0.5, 0.5	–	−
	26	−	2	2	2, 2, 2, 2	−	0, 0	–	−
13	16	−	2	2	2, 3, 2, 2	−	0, 1	–	−
	17	−	2	2	2, 2, 2, 3	−	1, 1	–	−
14	25	−	3	2	2,1,1,2	−	0, 0.5	–	−
Total/mean:	30	0	2.0 mm (1–3 mm)	1.8 mm (1–3 mm)	2.5 mm (1–5 mm)	13.3% of implants	0.5 mm (0–1.5 mm) mesially: 0.4 mm; distally: 0.6 mm	13.3% of implants	0

*mb* mesio-buccal, *db* disto-buccal, *mo* mesio-oral, *do* disto-oral, + present, − absent

## Discussion

In the present retrospective study, dental implants of reduced length (7 mm) that were placed in the posterior maxilla to avoid sinus augmentation procedure were clinically and radiologically followed up after a mean loading period of 5 years. The clinical and radiological results demonstrate successful midterm results regarding implant survival and peri-implant hard and soft tissue health. Low levels of bleeding on probing and the probing pocket depths indicate the absence of acute or chronical peri-implantitis and are therefore in accordance with the low values of peri-implant bone loss observed. Furthermore, the obtained favorable clinical and radiological results seemed to be independent of the applied prosthetic rehabilitation, as both, fixed and removable and splinted and un-splinted prosthetics, did not show any difference in clinical and radiological results.

Although bone augmentation procedures, such as sinus augmentation procedures, are frequently performed and well researched, patient demands tend to indicate minimally invasive treatments and reduced treatment periods [11]. The technical progress in implant materials and design over the past decades led to an expansion of the available implant diameters and lengths and, consequently, increased the ability to replace missing teeth, even in patients with reduced alveolar ridge dimensions. However, the acceptance of implants with reduced length, and, therefore, often an increased implant-crown ratio and associated adverse loading forces, is still reduced compared to standard-length implants. Research of the literature shows that most of these concerns are unfounded. Randomized clinical trials as well as systematic reviews show comparable clinical mid- and even partial long-term results when comparing “short implants” and conventional implants placed in combination with augmentative procedures. In a randomized multicenter study, the efficacy of short (5 or 6 mm long) dental implants compared to 10 mm or longer implants placed in crestally lifted sinuses indicated no significant differences regarding prosthesis and implant failures, complications, and radiographic peri-implant marginal bone level changes after a follow-up period of 3 years [12].

In a systematic review, Lemos et al. compared short implants with a length of 8 mm or less to standard implants (larger than 8 mm) placed in posterior regions of the maxilla and mandible. The authors reviewed 13 studies with a total of 1269 patients who had received a total of 2631 dental implants. Short implants showed marginal bone loss, prosthetic failures, and complication rates similar to those of standard implants. Therefore, short implants have been considered a reliable treatment for posterior jaws, especially in patients who require complementary surgical procedures [13].

In addition to biological complications, such as marginal bone loss, an increased crown-implant ratio has been suggested to increase the risk for technical complications of implant components. In an in vitro investigation, the influence of increased crown-to-implant ratios and mechanical stress on fracture and screw loosening was investigated. It was shown that an increased crown-implant ratio does not positively correlate with more frequent technical complications such as screw loosening [14].

Within the limits of the present single-arm study, such as the limited number of patients (14) and the midterm observation period of 5 years, implants with a length of 7 mm are suitable to replace missing teeth in the posterior maxilla and avoid sinus augmentation procedures. Though the literature reports predominantly satisfying results, further performance of prospective multicenter studies with high scientific evidence seems to be important to overcome the continued concerns about increased technical and biological susceptibility.

## Conclusion

The present retrospective study analyzed the clinical and radiological performance of dental implants of 7-mm length in the posterior maxilla used to avoid sinus augmentation procedures. After a mean period of loading of 5 years, a survival rate of 100% and an absence of peri-implant infections were detected, which leads to the conclusion that “short implants” are a reliable treatment option to avoid sinus augmentation procedures and replace missing teeth in the posterior maxilla.

## Abbreviations

−: Absent
+: Present
BoP: Bleeding on probing
db: Disto-buccal
do: Disto-oral
f: Female
f.p.: Fixed prosthetics
m: Male
mb: Mesio-buccal
MBL: Marginal bone loss
mo: Mesio-oral
PPD: Probing pocket depth
r.p.: Removable prosthetics

## References

[1] Lorenz J, Barbeck M, Kirkpatrick CJ, Sader R, Lerner H, Ghanaati S (2018). Injectable bone substitute material on the basis of β-TCP and hyaluronan achieves complete bone regeneration while undergoing nearly complete degradation. Int J Oral Maxillofac Implants.

[2] Lorenz J, Kubesch A, Korzinskas T, Barbeck M, Landes C, Sader R, Kirkpatrick CJ, Ghanaati S (2015). TRAP-positive multinucleated giant cells are foreign body giant cells rather than osteoclasts: results from a split-mouth study in humans. J Oral Implantol.

[3] Lorenz J, Korzinskas T, Poju C, Al Maawi S, Eichler K, Sader R, Ghanaati S (2018). Do clinical and radiological assessments contribute to the understanding of a biomaterials? Results from a prospective randomized sinus augmentation split-mouth trial. J Oral Implantol.

[4] Lorenz J, Kubesch A, Schwarz F, Sader RA, Schlee M, Ghanaati S. Contribution of allogeneic bone blocks to bone tissue regeneration in relation to their partially purification process - histological and histomorphometrical investigation. J Clin Oral Investigation. 2018; [Epub ahead of print]. 10.1007/s00784-018-2407-0 29524026

[5] Mangano FG, Shibli JA, Sammons RL, Iaculli F, Piattelli A, Mangano C (2014). Short (8-mm) locking-taper implants supporting single crowns in posterior region: a prospective clinical study with 1-to 10-years of follow-up. Clin Oral Implants Res.

[6] Atieh MA, Zadeh H, Stanford CM, Cooper LF (2012). Survival of short dental implants for treatment of posterior partial edentulism: a systematic review. Int J Oral Maxillofac Implants.

[7] Sun HL, Huang C, Wu YR, Shi B (2011). Failure rates of short (≤ 10 mm) dental implants and factors influencing their failure: a systematic review. Int J Oral Maxillofac Implants.

[8] Blanes RJ (2009). To what extent does the crown-implant ratio affect the survival and complications of implant-supported reconstructions? A systematic review. Clin Oral Implants Res.

[9] Nunes M, Almeida RF, Felino AC, Malo P, de Araújo Nobre M (2016). The influence of crown-to-implant ratio on short implant marginal bone loss. Int J Oral Maxillofac Implants.

[10] Lorenz J, Lerner H, Sader R, Ghanaati S (2017). Investigation of peri-implant tissue conditions and peri-implant tissue stability in implants placed with simultaneous augmentation procedure: a 3-year retrospective follow-up analysis of a newly developed bone level implant system. Int J of Impl Dent.

[11] Del Fabbro M, Rosano G, Taschieri S (2008). Implant survival rates after maxillary sinus augmentation. Eur J Oral Sci.

[12] Gastaldi G, Felice P, Pistilli R, Barausse C, Trullenque-Eriksson A, Esposito M (2017). Short implants as an alternative to crestal sinus lift: a 3-year multicentre randomised controlled trial. Eur J Oral Implantol.

[13] Lemos CA, Ferro-Alves ML, Okamoto R, Mendonça MR, Pellizzer EP (2016). Short dental implants versus standard dental implants placed in the posterior jaws: a systematic review and meta-analysis. J Dent.

[14] Bulaqi HA, Mousavi Mashhadi M, Safari H, Samandari MM, Geramipanah F (2015). Effect of increased crown height on stress distribution in short dental implant components and their surrounding bone: a finite element analysis. J Prosthet Dent.

